# Simultaneous assessment of colon motility in children with functional constipation by cine-MRI and colonic manometry: a feasibility study

**DOI:** 10.1186/s41747-021-00205-5

**Published:** 2021-02-10

**Authors:** M. H. Vriesman, C. S. de Jonge, S. Kuizenga-Wessel, B. Adler, A. Menys, A. J. Nederveen, J. Stoker, M. A. Benninga, C. Di Lorenzo

**Affiliations:** 1 Department of Pediatric Gastroenterology and Nutrition, Emma Children’s Hospital/Amsterdam UMC, University of Amsterdam, Amsterdam, The Netherlands; 2 Department of Radiology and Nuclear Medicine, Amsterdam UMC, Location Academic Medical Center, University of Amsterdam, Amsterdam Gastroenterology & Metabolism, Amsterdam, The Netherlands; 3 Department of Radiology, Nationwide Children’s Hospital, Columbus, OH USA; 4 Motilent, London, UK; 5 Department of Radiology and Nuclear Medicine, Amsterdam UMC, Location Academic Medical Center, University of Amsterdam, Amsterdam, The Netherlands; 6 Department of Radiology and Nuclear Medicine, Amsterdam UMC, Location Academic Medical Center, University of Amsterdam, Amsterdam Gastroenterology & Metabolism, Cancer Center Amsterdam, Amsterdam, The Netherlands; 7 Division of Gastroenterology, Hepatology and Nutrition, Nationwide Children’s Hospital, Columbus, OH USA

**Keywords:** Child, Colon, Constipation, Magnetic resonance imaging (cine), Manometry

## Abstract

**Background:**

Colonic manometry is the current reference standard for assessing colonic neuromuscular function in children with intractable functional constipation (FC). Recently, cine magnetic resonance imaging (cine-MRI) has been proposed as a non-invasive alternative. We compared colonic motility patterns on cine-MRI with those obtained by manometry in children, by stimulating high-amplitude propagating contractions (HAPCs) with bisacodyl under manometric control while simultaneously acquiring cine-MRI.

**Methods:**

After Institutional Review Board approval, adolescents with FC scheduled to undergo colonic manometry were included. A water-perfused 8-lumen catheter was used for colonic manometry recordings. After an intraluminal bisacodyl infusion, cine-MRI sequences of the descending colon were acquired for about 30 min simultaneously with colonic manometry. Manometry recordings were analysed for HAPCs. MRI images were processed with spatiotemporal motility MRI techniques. The anonymised motility results of both techniques were visually compared for the identification of HAPCs in the descending colon.

**Results:**

Data regarding six patients (three males) were analysed (median age 14 years, range 12–17). After bisacodyl infusion, three patients showed a total of eleven HAPCs with colonic manometry. Corresponding cine-MRI recorded high colonic activity during two of these HAPCs, minimal activity during seven HAPCs, while two HAPCs were not recorded. In two of three patients with absent HAPCs on manometry, colonic activity was recorded with cine-MRI.

**Conclusions:**

Simultaneous acquisition of colonic cine-MRI and manometry in children with FC is feasible. Their motility results did not completely overlap in the identification of HAPCs. Research is needed to unravel the role of cine-MRI in this setting.

## Key points

- Simultaneous acquisition of colonic cine magnetic resonance imaging (cine-MRI) and manometry in children with is feasible.
- Agreement between colonic motility results of manometry and cine-MRI was low.
- The role of colonic cine-MRI in children with functional constipation should be investigated in larger patient cohorts.

## Background

Functional constipation is a worldwide common health disorder in children [1]. The diagnosis of functional constipation is made according to the symptom-based Rome criteria [2, 3] obtained from medical history and physical examination. Most children with functional constipation respond well to conventional therapeutic strategies, including behavioural interventions and laxative treatment. However, a subset of children with severe constipation remains unresponsive to these treatments and may require additional testing [4]. Colonic manometry has been shown to be a valuable diagnostic test in the differentiation between normal colonic motility patterns, which are present in functional constipation, *versus* colonic neuromuscular disorders [5]. Accurate assessment of colonic motility can be essential in the work-up of intractable functional constipation and may serve as a useful guide for medical and surgical strategies [5]. Interpretation of colonic manometry recordings involves the identification of high-amplitude propagating contractions (HAPCs) after a meal or chemically induced by stimulants such as bisacodyl. HAPCs are colonic motor patterns responsible for movements of bowel contents in an anterograde direction and are associated with defecation [6, 7]. The presence of HAPCs is one of the indicators of normal colonic motility [5].

Although colonic manometry has been found to be tolerated in most children, it requires a costly hospital admission with bowel clean-out and catheter placement under sedation or general anaesthesia. Moreover, manometry is not widely available, with only a few paediatric centres worldwide performing the examination routinely in children.

Therefore, other techniques such as magnetic resonance imaging (MRI) have been advocated as a less invasive imaging technique for the characterisation of gastrointestinal motility. Emerging literature reports that so-called “cine” MRI techniques may safely assess gut motility for a variety of gastrointestinal disorders in both children and adults [8–12]. One study in healthy adults showed that cine-MRI allows for visualisation and reliable recording of HAPCs as compared to colonic manometry [13]. To date, data on the use of this technique in children with intractable functional constipation have yet to be obtained.

Therefore, the aim of this feasibility study was to compare the identification of colonic motility patterns on cine-MRI with those obtained by manometry in children with functional constipation, by stimulating high-amplitude propagating contractions (HAPCs) with bisacodyl under manometric control while simultaneously acquiring cine-MRI.

## Methods

### Participants

Between May 2012 and June 2013, all children with intractable functional constipation aged 12-to-18 years, scheduled to undergo colonic manometry recordings at the Motility Department of the Division of Gastroenterology, Nationwide Children’s Hospital, Columbus, OH, USA, were invited to participate. All patients fulfilled the Rome III criteria [2] for functional constipation and had long lasting symptoms of constipation and faecal incontinence. Exclusion criteria were general contraindications for MRI scanning (*e.g.*, claustrophobia and metal implants such as ferromagnetic surgical clips and pins) and the use of antidepressant drugs or drugs interacting with the central nervous system in the previous 5 days. The study was approved by the local Institutional Review Board. Written informed consent was obtained from each patient and parent.

### Study protocol

Our study protocol resembled the study of Kirchhoff et al. [13]. To compare the identification of HAPCs, children underwent colonic manometry and simultaneous cine-MRI recordings after the administration of a motility-inducing drug (bisacodyl). First, children had colonic manometry recordings at the Motility Department for a minimum of 60 min during fasting and 60 min after a meal. Patients were then moved to the MRI department for simultaneous cine-MRI recordings. After the patient was moved to the MRI department, the cine-MRI examination in parallel with the manometry data acquisition was started with a survey scan. After this survey scan, 10 mg of bisacodyl (Dulcolax, Boehringer Ingelheim, Biberach, Germany) suspended in 30 mL 0.9% saline was administered intraluminally via the open tip of the manometry catheter to stimulate contractions. For children with a caecostomy, the bisacodyl solution was intraluminally administered via a tube placed through the stoma. After bisacodyl infusion, cine-MRI images of the descending colon were obtained for about 30 min while colonic manometry recordings continued simultaneously. After cine-MRI was completed, patients were transported back to the Motility department, where the last phase of the manometry recordings continued. The MRI images were stored on a Picture Archive and Communication System (General Electric Healthcare, Milwaukee, WI, USA), manometry recordings were electronically stored using a validated software (Medical Measurement Systems, MMS, version W8.17f, Enschede, The Netherlands).

### Colon manometry protocol and motility analysis

The day before the study, patients were prepared with polyethylene glycol electrolyte lavage solution, administered via nasogastric tube. Motility-inducing medications were stopped 48 h prior until motility recordings were completed. The manometry catheter was placed either by routine colonoscopy under general anaesthesia or by the interventional radiologist under sedation, guided by fluoroscopic control [14]. A water-perfused 8-lumen catheter (Mui Scientific, ON, Canada), with recording sites every 10 cm and an open tip, was placed in the colon for motility recordings. To allow for MRI recordings, the catheter was not clipped onto the colonic mucosa. An abdominal x-ray was performed to check the position of the catheter. After patients had fully recovered from the anaesthesia or sedation, the colonic manometry recordings were carried out on the same day. The expected effect of anaesthesia or sedation on colonic motility, several hours before starting the simultaneous colonic manometry and cine-MRI recordings, was considered neglectable.

**Fig. 1:**
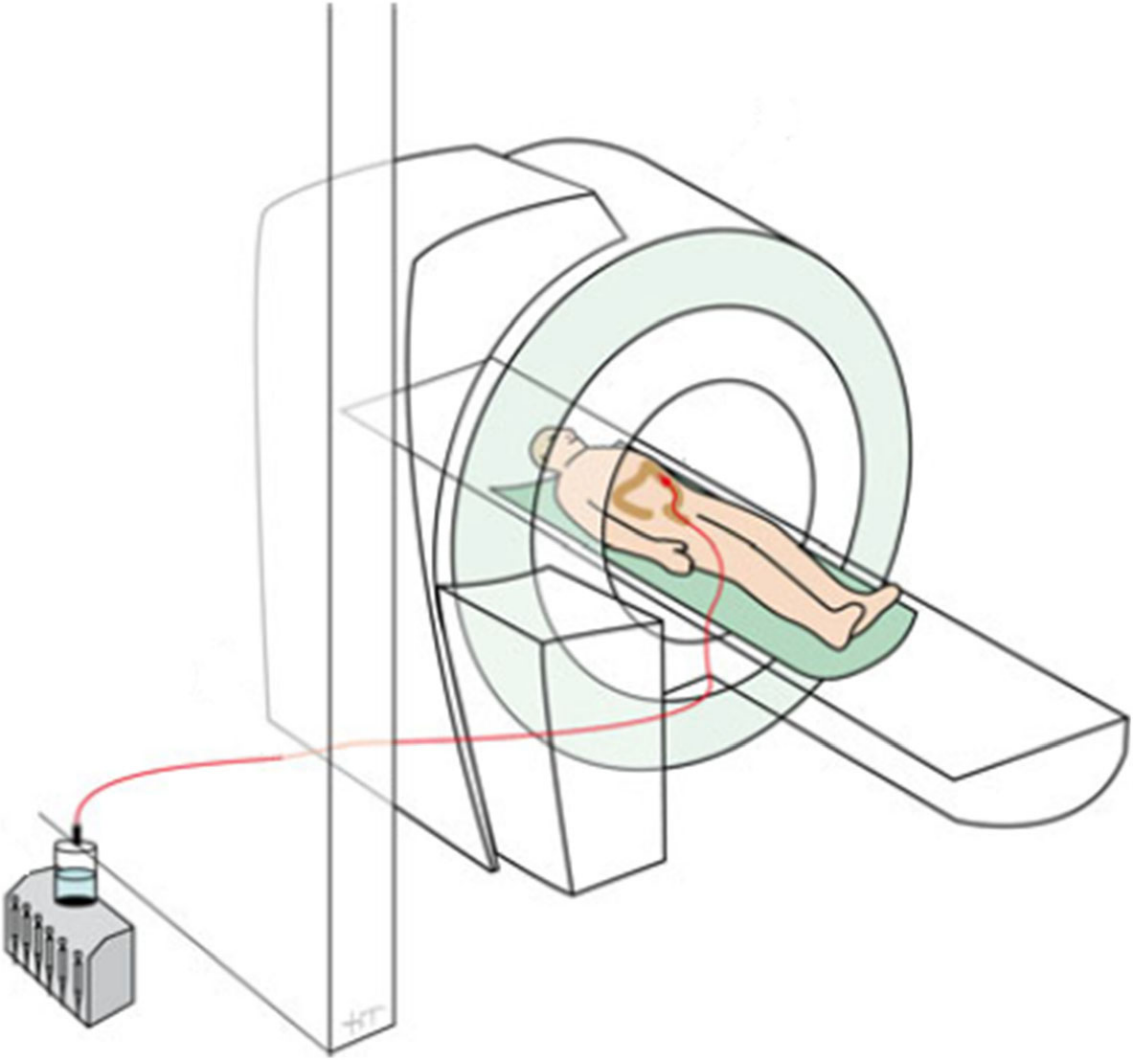
Study setup simultaneous motility recordings using cine magnetic resonance imaging (MRI) and colonic manometry. Colonic manometry catheter was connected to a pneumohydraulic pump outside the MRI scanner room. Reprint with permission from [13]

The length of the manometry catheter was extended to 300 cm to allow performing MRI. The catheter was connected outside the MRI scanner room because the transducers of the manometry device were not MRI safe and would break inside the MRI room due to the strong magnetic field (Fig. 1). Outside the MRI scanner room, the manometry catheter was connected to a water perfusion system with a low-compliance pneumohydraulic pump. Data were recorded on a multichannel system (MMS, Enschede, The Netherlands). As per our manometry protocol, patients were confined to bed until the manometry recordings were completed to guard against dislodgement of the catheter. A repeat abdominal x-ray was performed in cases of catheter dislodgement or if the recording tracing suggests coiling of the catheter in the rectum.

To assess colonic motility, focused specifically on the descending colon, manometric recordings were visually analysed for pressure changes to the recording channels of the catheter (in mmHg) by two experienced paediatric gastroenterologists (C.L., A.G.). The number and the point in time of HAPCs stimulated after bisacodyl administration were determined using the already mentioned software (MMS, version W8.17f). HAPCs were defined as clusters of contractions with an amplitude ≥ 60 mmHg and antegrade propagation over at least three adjacent recording sites [15].

### Cine-MRI protocol and motility analysis

The MRI images were acquired using a 1.5-T MRI scanner (Signa HDxt platform, software version LX HD16, General Electrci Healthcare, Milwaukee, WI, USA) in supine position. Colon motility scans were obtained using a sagittal T2-weighted single-shot fast spin-echo sequence with following technical parameters: echo time 90 ms; repetition time 1,150 ms, flip angle 90°, field of view 300 × 400 mm, slice thickness 4 mm, temporal resolution 1 frame/s). The sequence was chosen because of the short acquisition time and good air/soft tissue contrast [16]. The slice location was chosen aiming at the longitudinal direction of the descending colon. The motility scan was acquired during about 30 min of free breathing. Patients were not sedated and were asked to breathe quietly throughout the image acquisition. No oral bowel preparation was administered before scanning. Additionally, children were allowed to watch a movie.

Colonic motility on the cine-MRI was captured in the descending colon. Images were processed with a spatiotemporal motility MRI analysis tool (Motilent, London, UK). As previously described [17, 18], this method automatically measures systematic, cross-sectional diameters in the gastrointestinal tract. In summary, the user draws a midline in the descending colon and delineates the walls of the colon, subsequently the software draws lines perpendicular to the midline. When the colon contracts (narrows and relaxes), the bowel walls move and the length of these lines change at that time point. Therefore, changes in the length of each line between consecutive time points are identified as colonic activity. Subsequently, residuals are calculated by fitting a line to the series of distances (per position), resulting in a two-dimensional spatiotemporal residuals plot corresponding to the changes in luminal diameter over time.

This motility plot is visualised in a similar way as a colonic manometry, with red colours representing a small diameter (*i.e.*, contraction, and throughout the manuscript referred to as ‘high’ colonic activity), blue colours representing no change in diameter (*i.e.*, no colonic activity), and yellow colours representing “minimal” colonic activity [16].

### Simultaneous recordings of cine-MRI and colon manometry

**Fig. 2:**
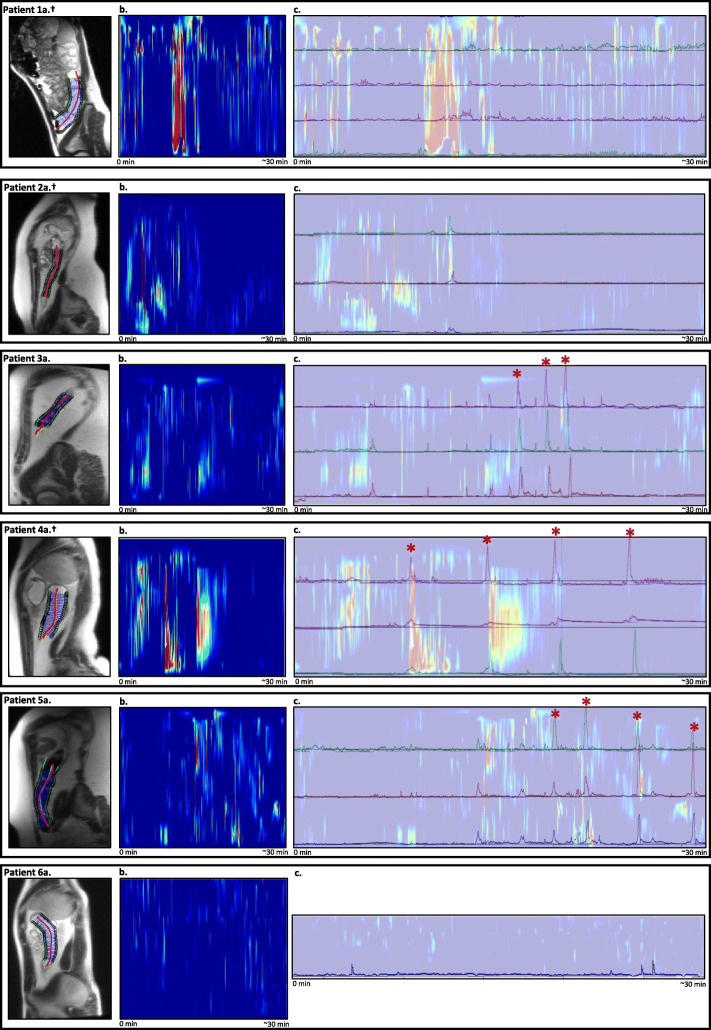
Simultaneous colonic manometry and cine magnetic resonance imaging (cine-MRI) motility results. The figure displays: (**a**) a sagittal view of the child showing the region of interest in the descending colon; (**b**) the results of about 30 min of cine-MRI colonic activity. Recording time in minutes is displayed on the *x*-axis. Colonic activity is reported on the *y*-axis, with red colours representing a small diameter (contraction) and blue colours no change in diameter; and (**c**) the about 30 min of colonic manometry recordings with an overlay of simultaneous cine-MRI results as shown in **b**, to allow for a visual comparison of high-amplitude propagating contractions (HAPCs). The *x*-axis displays the time from the start of simultaneous cine-MRI imaging (0 min) to the termination of the cine-MRI recordings (about 30 min). The *y*-axis displays the manometric channels in the descending colon with pressure changes (in mmHg), overlapping the cine-MRI motility results as shown in **b**. ± Patient with caecostomy in place, * HAPC

After the individual motility assessment by colonic manometry and cine-MRI, the anonymised results of both techniques were visually compared during the ~ 30 min of simultaneous recordings after bisacodyl infusion. For each individual patient, the manometric channels in the descending colon that were visualised on the cine-MRI images were identified. Next, an overlay between the two techniques was created (Fig. 2) and the cine-MRI results were visually analysed for corresponding motility during HAPCs as recorded on the manometry results. Only the manometric channels visible on MRI were used in the analysis.

## Results

### Participants

Twelve patients with intractable functional constipation scheduled to undergo colonic manometry agreed to participate in the study. Out of these twelve children, four patients were excluded due to preliminary termination of the colonic manometry recordings before the cine-MRI data was obtained (*i.e.*, patient withdrawal, toleration issues. or preliminary catheter expulsion). Two patients could not tolerate the MRI scanner due to claustrophobia. Therefore, overall, data from six patients were analysed (three males), with a median age 14 years (range 12–17 years) (Table 1). Three patients had a caecostomy in place, through which the colonic catheter was placed. These three children were unresponsive to high-doses oral and rectal laxatives and were therefore treated with antegrade administration of laxatives through a minimally invasive surgical created opening in the caecum.

**Table 1:** Colonic manometry data

Patient number/male or female	Additional clinical information	Current management	Position tip colonic catheter (as shown on abdominal x-ray)	Colonic manometry channels visualised on cine-MRI	Number of HAPCs visualised on manometry in the descending colon	Results of colonic manometry
1/F	Concomitant symptoms of abdominal pain, nausea and depression	Treated with daily caecostomy flushes with saline and polyethylene glycol	Sigmoid colon	3-6	0	Abnormal colonic motility with lack of HAPCs in response to the administration of bisacodyl
2/F	Concomitant symptoms of gastroesophageal reflux disease	Treated with daily caecostomy flushes with saline, bisacodyl and polyethylene glycol	Distal descending colon	6–8	0	Normal colonic motility in the colon proximal to the splenic flexure; abnormal motility in the descending colon with lack of HAPCs in response to the administration of bisacodyl
3/M	Known with obesity	Treated with high dosage of daily lactulose, polyethylene glycol and bisacodyl	Hepatic flexure	5–7	3	Normal colonic motility
4/F		Treated with daily caecostomy flushes with saline and bisacodyl	Rectosigmoid colon	4–6	4	Normal colonic motility
5/M	Known with obesity	Weekly cleanout with high dose polyethylene glycol and daily bisacodyl.	Cecum	6–8	4	Normal colonic motility
6/M	Known with attention deficit/hyperactivity disorder	Daily polyethylene glycol	Hepatic flexure	8	0	Abnormal colonic motility with lack of HAPCs in response to the administration of bisacodyl

*HAPCs* High-amplitude propagating contractions. The age of individual patients is not given avoiding identification

### Simultaneous cine-MRI and colon manometry

In total, three out of six included patients had visual HAPCs on the manometric recordings in the descending colon (Table 1). Corresponding colonic activity of the descending colon of each individual patient was visually compared with the cine-MRI results and displayed in Fig. 2.

#### Study patient 1

Study patient 1 was a girl with a caecostomy in place, through which the colonic catheter was inserted. Tip of the catheter was placed in the sigmoid colon and four channels of the colonic manometry were recorded on the cine-MRI. After bisacodyl infusion, data of 32 min of simultaneous MRI and manometry recordings were collected. No HAPCs were recorded during the colonic manometry recordings. Cine-MRI showed one episode of high activity, corresponding to minimal pressure changes on the colonic manometry recordings (Fig. 2(1c)).

#### Study patient 2

Study patient 2 was a girl with a caecostomy. The colonic catheter was placed into the distal descending colon and three channels were recorded on the cine-MRI. No HAPC was recorded on the manometry recordings in the descending colon. Minimal episodes of activity were recorded with cine-MRI (Fig. 2(2c)).

#### Study patient 3

Study patient 3 was a male. Tip of the colonic catheter was placed in the hepatic flexure, and three channels were recorded on the cine-MRI. After bisacodyl infusion, three HAPCs were measured with manometric recordings. Minimal episodes of activity were recorded with cine-MRI (Fig. 2(3c)).

#### Study patient 4

Study patient 4 was a girl with symptoms of intractable functional constipation and faecal incontinence and had a caecostomy. Tip of the catheter was placed in the rectosigmoid colon and three channels were recorded on the cine-MRI. After intraluminal bisacodyl infusion, four HAPCs were recorded in the descending colon. The corresponding cine-MRI recorded three episodes of high activity (Fig. 2(4c)). Two of these high activity episodes overlapped with the HAPCs as recorded on the manometry, two HAPCs were not recorded with cine-MRI. One high activity episode recorded with cine-MRI corresponded to minimal pressure changes on the colonic manometry recordings.

#### Study patient 5

Study patient 5 was a male child, with the tip of the colonic catheter placed in the caecum. Three channels were recorded on the cine-MRI images. A total of four HAPCs were visualised on the manometry recordings in the descending colon. During these HAPCs, the corresponding cine-MRI recorded very minimal episodes of activity (Fig. 2(5c)).

#### Study patient 6

Study patient 6 was a male with the tip of the catheter placed at the hepatic flexure. One channel was visual on the cine-MRI. During the 27 min of simultaneous measuring after bisacodyl infusion, no HAPCs were recorded with manometry and no activity was recorded with corresponding cine-MRI (Fig. 2(6c)).

### Agreement detection HAPCs between colonic manometry and cine-MRI

Manometric recordings identified a total of eleven HAPCs (Table 1). Two HAPCs (2/11 = 18%) were clearly recorded with cine-MRI and two HAPCs (18%) were not recorded. During the other 7/11 HAPC events (64%), minimal activity was only recorded with cine-MRI.

Three patients had no evidence of HAPCs on the colonic manometry recordings, corresponding cine-MRI recordings showed absent colonic activity in one patient (patient 6), but showed minimal activity in the other patients (patients 1 and 2). In patient 1, there was one major colonic activity episode recorded with the cine-MRI, but no motility was recorded with manometry.

## Discussion

This feasibility study shows the use of cine-MRI for colonic motility analysis, in direct comparison with colonic manometry. It is the first study to describe a novel semi-automatic cine-MRI motility analysis technique in children with functional constipation. Our results demonstrate that the cine-MR-imaging technique was able to detect colonic activity when manometric recordings measured HAPCs; however, the agreement between colon manometry results and cine-MRI motility results was low.

Better understanding of colonic motility patterns could benefit children suffering from intractable functional constipation, because patients with evidence of (partial) colonic dysmotility may require a different management than patients with normal motility patterns [19, 20]. Accurate assessment of motility is therefore essential in the (surgical) therapeutic strategies of these children. Over the past 25 years, colonic manometry has become a valuable tool in the management of children with intractable constipation [5]. An advantage of this technique is the possibility for long durations of measurements in the range of hours, a major limitation however is that it is an invasive procedure that requires an extensive bowel preparation and children are confined to bed for several hours, representing non-physiological conditions [21]. In addition, the intraluminal pressure changes measured with colonic manometry may not always correlate with colonic contractions or movement of colonic contents [22]. Hence, cine-MRI has been proposed as an alternative to assess colonic motility. In contrast to manometry, MRI is a non-invasive technique, widely available and costs may be relatively low due to the shorter duration of scanning and avoidable bowel clean out and anaesthesia [8]. However, during a cine-MRI scan, patients are required to lay still for several minutes in a tight spaced scanner; therefore, compliance is argued to be challenging in (young) children. Indeed, in our cohort, two out of twelve children were not able to tolerate the MRI scanner due to symptoms of claustrophobia. Nevertheless, it is important to keep in mind that MRI scanning is already widely implemented in paediatric clinical care and compliance can be improved with appropriate education of patients and radiology staff, including behavioural techniques and audio-visual support devices [23].

Our study design was based on a previous study of Kirchhoff et al. [13], conducted in 2010. This study included ten healthy adults and found 100% sensitivity in identifying HAPCs with MRI compared to colonic manometry. We could not confirm these promising results in our study. Several factors could play a role in the differences found between the two studies. First, instead of healthy adults, we included children, who were also suffering from severe functional constipation. To date, it remains unclear whether manometry results from adult studies can be extrapolated to the paediatric population and *vice versa* because colonic manometry samples in healthy children are lacking as a reference. However, similar to adults, it has been recognised that colonic motor activity is reduced in children with functional constipation [24–26]. Therefore, in this specific patient group, it was not surprising that it was challenging to identify aspects of normal motility such as HAPCs. Another possible explanation for the differences found between the studies could be the lower temporal resolution of the MRI sequence used in our study, further discussed below. In addition, since 2010, major developments have been made in cine-MRI acquisition and quantification techniques. Therefore, we were able to use a novel semi-automated cine-MRI analysing technique [18], enabling a more detailed manner of colonic wall movement assessment, possibly contributing to the differences in results.

Comparing two independent techniques introduces data alignment challenges when they are founded on two distinct physics principles like manometry and cine-MRI. With water-perfused colonic manometry, motility is represented by intraluminal pressure changes. Contractions of the colonic wall may temporarily occlude the ports of the inserted manometric catheter, leading to resistance in water flow, which in turn is registered as an increase in intraluminal pressure [21]. With the cine-MRI technique, colonic motility is represented by a metric based on changes in diameter of the colonic wall over time at a given position [18]. One can conclude that a measure of pressure is not directly equivalent to a change in colonic wall diameter; therefore, deviations between the measurements can be expected.

In addition, we note that not all HAPCs observed through manometry recordings were identified with cine-MRI. *Vice versa*, in two of three patients, motility was measured with cine-MRI while HAPCs were absent on the colonic manometry recordings. It could be hypothesised that cine-MRI was more sensitive in detecting low-amplitude colonic motility compared to the pressure sensors of the manometry. This is supported by a recent work by Menys et al. [18] using a similar cine-MRI analysing technique for colonic motility assessment in 20 healthy adults. Their results reported that the cine-MRI was able to detect even minor changes in colonic wall diameter, potentially representing small localised contractions [18]. But it could also (partly) be explained by the following limiting factors. Our data was acquired in 2012-2013, however novel cine-MRI analysing techniques (such as spatiotemporal motility analysis) enabled innovative ways in 2019.

Moreover, colonic manometry was performed using a water-perfused manometry catheter. These low-resolution catheter types are shown to reliably identify large motor patterns, but they are proven to have difficulties in detecting motor activity other than HAPCs [27, 28], especially when the pressure sensors are spaced wider apart (> 3 cm) [29]. Our cine-MRI sequences had a low temporal resolution, with images obtained every 6 s. Changes in colonic diameter between two consecutive images could therefore have been missed, possibly resulting in underreporting of colonic motility information. This could explain why not all HAPCs on the manometric recordings were identified by the cine-MRI or only minimally visualised.

Another limitation of the obtained cine-MRI data is that the images were obtained in two-dimensional sagittal orientation with limited field of view. It could be hypothesised that during high-amplitude contractions, the colon moves (partly) out of the examination plane and was therefore not visualised on the MRI scan. In that case, the measured colonic diameter was smaller, mimicking a contraction, but in reality it was a displacement that was captured in the measurement. Acquiring three-dimensional (3D) cine-MRI datasets with coverage of the total colon in combination with a 3D quantification method would solve this issue. Unfortunately, acquiring 3D cine-MRI imaging with coverage of the total colon, while at the same time acquiring the images at a high enough temporal resolution is very challenging. At this moment in time, the 3D cine-MRI acquisition techniques are improved compared to the date of our data collection, but this topic is an on-going focus of research. Furthermore, owing to the current developmental status of the MRI analysing techniques, a clear definition of HAPCs measured by MRI is lacking and the amplitude of the contraction was not taken into consideration in the study. Therefore, with the lack of comparison cine-MRI data in children, we were unable to define normative values for colonic activity and the cine-MRI plot was only described visually. Although ethically challenging in healthy children, future studies on the use of cine-MRI in children without constipation are needed to gain more insight on normative data.

In contrast to the contractility of the heart, motor patterns of the colon are usually unpredictable and occur less frequently, thus requiring longer MRI data acquisition during free breathing [30]. Especially after a bowel cleanout, eliminating the physiologic stimulant for propagating contractions, HAPCs are rare events, even in healthy individuals [31]. It could therefore be argued that the about 30 min of cine-MRI data acquisition in patients with functional constipation could be a too short timeframe to reliably identify colonic motor patterns. We therefore chose to identify HAPCs with simultaneous assessment of colonic manometry and cine-MRI after the intraluminal infusion of bisacodyl, which is known to induce HAPCs similarly to physiological occurring HAPCs in children [32]. A previous study in children with intractable constipation showed a mean time interval between the bisacodyl administration and the first HAPC of 4 min (range 1–8) [24]. Therefore, the duration of parallel cine-MRI acquisition should be sufficient.

Registration between manometry and cine-MRI data was challenging due to the used protocol, this may have contributed to the non-agreement of the colonic manometry and cine-MRI. Therefore, with the use of older manometric catheter types, MRI scanner and acquisition techniques in this study, caution should be taken when formulating a conclusion on the detection of low-amplitude colonic motility.

Strengths of the study include the use of simultaneous motility recordings and blinded data analysis, making the study less susceptible for bias. General limitations regarding the study were the small sample size of six children, including patients with and without caecostomy and different bisacodyl administration sites, making it challenging to formulate strong uniform conclusions. However, data of 165 manometry studies in children with intractable constipation showed that the site of bisacodyl administration (through the stoma or catheter tip) did not alter the HAPC response [33]. In one child (patient 6), only one channel was visualised on the MR-images and important data could therefore have been missed. Lastly, because a uniform protocol for additional diagnostic testing was lacking in the study (including transit time, anorectal manometry, and contrast enema), important information on the condition of the descending colon of included children was often missing and could not be correlated with the colonic motility results.

Since 2013, there have been several technical developments in the field of both colonic manometry and cine-MRI acquisition techniques, possibly contributing to a more thorough evaluation of colonic motility patterns. High-resolution manometry solid-state catheters became available, with pressure sensors every 1–2.5 cm instead of 10 cm as used in this study. These high-resolution manometry catheters are now able to detect more distinct colonic motor patterns in children, in particular that of low-amplitude motor activity [24, 34, 35]. Future research in children is needed to better understand our current clinical observations made with these novel manometry techniques.

The development and evaluation of cine-MRI acquisition and quantification techniques has been evolving. The spatiotemporal motility analysis technique is currently only available for research since it has been recently developed and its clinical value needs to be firstly assessed through research studies. The information gained from the current study contributes to the exploration of the value of the cine-MRI-based spatiotemporal motility analysis for its diagnostic use in children. Our study is a first step in the validation of cine-MRI against colonic manometry in children, but future studies are needed to unravel the potential role of cine-MRI gastrointestinal motility analysis. These studies should investigate the use of MRI safe high-resolution manometry catheters using high temporal resolution sequences. Studies should include a large sample size of children, ideally compared to a control group of children without gastrointestinal disorders, using a standardised protocol with clear definition of HAPCs as recorded on MRI images. A clinical trial using high-resolution manometry against cine-MRI is currently running in adults with constipation (NCT03226145, clinicaltrials.gov) [36]. In the future, we envisage a workflow where the two techniques work in a complementary fashion with MRI, perhaps being used to triage patients to manometry levering the respective strengths of the two techniques.

In conclusion, this feasibility study, including a small cohort of children with intractable functional constipation, showed that cine-MRI was able to record motility simultaneously with colonic manometry. However, the agreement between colonic motility results of the manometry and cine-MRI was low. Therefore, until future data is acquired, cine-MRI should be seen as a complementary technique to assess gastrointestinal motility rather than a substitute for colonic manometry.

## Data Availability

Not applicable (data for each individual patient are reported in this manuscript).

## Abbreviations

3D: Three-dimensional
HAPCs: High-amplitude propagating contractions
MRI: Magnetic resonance imaging
